# Allogeneic Hematopoietic Stem Cell Transplantation in Extranodal Natural Killer/T-cell Lymphoma

**DOI:** 10.4274/tjh.galenos.2021.2020.0438

**Published:** 2021-06-01

**Authors:** Yin-yin Peng, Yi-ying Xiong, Li-xia Zhang, Jing Wang, Hong-bin Zhang, Qing Xiao, Shu-liang Guo

**Affiliations:** 1 First Affiliated Hospital of Chongqing Medical University, Department of Hematology, Chongqing, China; 2 First Affiliated Hospital of Chongqing Medical University, Department of Respiratory Medicine, Chongqing, China

**Keywords:** Extranodal NK/T-cell lymphoma, Chemotherapy, Radiotherapy, Allogeneic hematopoietic stem cell transplantation

## Abstract

**Objective:**

Extranodal NK/T-cell lymphoma (ENKL) is aggressive and resistant to chemotherapy and radiotherapy. Allogeneic hematopoietic stem cell transplantation (allo-HSCT) is a potentially curative treatment for high-risk lymphomas owing to its associated graft-versus-lymphoma (GVL) effect. However, its application to ENKL is limited. We aim to summarize the characteristics of allo-HSCT for ENKL and, more importantly, evaluate whether allo-HSCT could offer any benefits for ENKL.

**Materials and Methods:**

A systematic review and data analysis were performed to evaluate the performance of allo-HSCT in the treatment of ENKL using studies obtained from PubMed, Medline, and Embase from January 2000 to December 2019 in the English language.

**Results:**

A total of 136 cases from 17 eligible publications were included in this study. It was found that after allo-HSCT, with an average follow-up time of 34 months (range: 1-121 months), 37.5% (52) of 136 patients had acute graft-versus-host disease (GVHD) and 31.6% (43) had chronic GVHD. Furthermore, 35.3% (48) of the patients were reported to have relapsed, but 2 of those relapsed only locally and achieved complete remission (CR) again with additional irradiation, chemotherapy, and donor lymphocyte infusions for one and rapid tapering and discontinuation of cyclosporine for the other, earning more than one year of extra survival. Finally, of the 136 patients, 51.5% (70) died because of primary disease progression (42.9%), infection (20.0%), GVHD (11.4%), organ failure (7.1%), hemorrhage (4.3%), and other causes (not specified/unknown) (14.3%).

**Conclusion:**

Allo-HSCT may be a treatment option for advanced or relapsed/refractory ENKL, but its role still requires more rigorous future studies.

## Abstract (tr)

**Amaç:**

Ekstranodal NK/T-hücreli lenfoma (ENKL) agresiftir ve kemoterapi ve radyoterapiye dirençlidir. Allojenik hematopoetik kök hücre transplantasyonu (allo-HSCT), ilişkili graft-lenfoma (GVL) etkisi nedeniyle, yüksek riskli lenfomalara yönelik potansiyel olarak iyileştirici bir tedavidir. Bununla birlikte, ENKL’ye uygulanması sınırlıdır. Bu çalışmada ENKL için allo-HSCT’nin/AHKHN’nin özelliklerini özetlemeyi ve daha da önemlisi allo-HSCT’nin ENK için herhangi bir fayda sağlayıp sağlamayacağını değerlendirmeyi amaçlıyoruz.

**Gereç ve Yöntemler:**

Ocak 2000’den Aralık 2019’a kadar İngilizce dilinde PubMed, Medline ve Embase literatürleri kullanılarak allo-HSCT’nin ENKL’ye performansını değerlendirmek için sistematik bir inceleme ve veri analizi gerçekleştirildi.

**Bulgular:**

Bu çalışmaya 17 uygun yayından toplam 136 olgu dahil edildi. 1) allo-HSCT’den sonra, 34 aylık ortalama takip süresine göre (aralık: 1-121 ay), 136 hastanın %37,5’inde (52) akut graft-versus-host hastalığı (GVHD), %31,6’sında (43) kronik GVHD vardı; 2) rapor edildiğinde, hastaların %35,3’ünde (48) relaps vardı, ancak bunlardan ikisi sadece lokal olarak nüks etti ve bunlardan birine ek ışınlama, kemoterapi, donör lenfosit infüzyonu ile tekrar tam remisyon (CR) sağladı, diğerine siklosporinin hızlı azaltılması ve kesilmesi bir yıldan fazla ekstra sağkalım kazandırdı; 3) 136 hastanın %51,5’i (70) primer hastalık ilerlemesi (%42,9), enfeksiyon (%20,0), GVHD (%11,4), organ yetmezliği (%7,1), kanama (%4,3) ve diğerleri (belirtilmedi/bilinmiyor) (%14,3) nedeniyle öldü.

**Sonuç:**

Allo-HSCT, ilerlemiş veya nükseden/refrakter ENKL için bir tedavi seçeneği olabilir, ancak rolü hala daha titiz gelecek çalışmaları gerektirmektedir.

## Introduction

Natural killer cell tumors are categorized as extranodal NK/T-cell lymphoma (ENKL), aggressive NK-cell leukemia and chronic NK-cell lymphoproliferative disorders according to the 2016 World Health Organization (WHO) classification [1]. Among them, ENKL is relatively more common. ENKLs include both nasal and extra-nasal ENKL categories of disease in the current 2016 WHO classification [2]. It is rarely diagnosed in Western countries but relatively more common in East Asian countries, being closely associated with Epstein-Barr virus (EBV). Pathologically, ENKL shows a highly aggressive clinical behavior. It usually involves the nasal cavity, nasopharynx, upper aerodigestive tract, skin, gastrointestinal tract, or other parts of the body, which have poorer survival. Limited-stage ENKL responds relatively much better to radiotherapy or to concurrent radiation and chemotherapy than advanced or relapsed/refractory ENKL. So far, there is no standard management for relapsed or refractory disease.

Although ENKL’s clinical features and prognostic factors have been well characterized in the last decades, optimal treatment strategies still remain unclear. The tumor cells lack L-asparagine synthetase and are susceptible to L-asparaginase, which depletes L-asparagine in NK lymphoma cells [1]. Regimens containing L-asparagine were effective for limited-stage ENKL; however, when used in patients with advanced-stage disease, these regimens were not so satisfactory. Some studies showed that the complete remission (CR) rate with the L-asparagine, etoposide, and dexamethasone (AspaMetDex) regimen for patients with advanced-staged disease was 30%, and the 5-year survival rate with the L-asparagine, vincristine, and dexamethasone (LVD) regimen was only 25% [3,4]. As advanced-stage or relapsed/refractory ENKL is highly progressive and sometimes multidrug-resistant, L-asparaginase-based therapy is still a challenge [5].

Allogeneic hematopoietic stem cell transplantation (allo-HSCT) is a potentially curative treatment for high-risk lymphoma patients owing to its associated graft-versus-lymphoma (GVL) effect. Furthermore, ENKL cells almost invariably express EBV antigens, providing an alloreactive target to enhance the GVL effect [1]. Although allo-HSCT is an effective way to treat hematologic tumors, its applications for ENKL remain limited. Some small series of studies have demonstrated a disease-free survival (DFS) of 30%-50%, but with high transplant-related mortality of about 25% [1,6,7,8,9]. Problems still remain: (1) undoubtedly, both infection and acute graft-versus-host disease (GVHD) during allo-HSCT often cause death; (2) the heavy psychological pressure during allo-HSCT is still overwhelming; (3) and, furthermore, human leukocyte antigen (HLA) donors and patients are still hard to match for allo-HSCT. Therefore, in this study we aim to summarize the characteristics of allo-HSCT for ENKL and, more importantly, evaluate whether allo-HSCT could offer any benefits for ENKL.

## Materials and Methods

### Literature Review

We searched PubMed, Medline, and Embase for publications in the English language from January 2000 to December 2019. The following terms were used: “natural killer/T-cell tumors” or “natural killer/T-cell neoplasm” or “natural killer/T-cell lymphoma” or “extranodal natural killer/T-cell lymphoma” or “extranodal natural killer/T-cell lymphoma nasal type” or “angiocentric lymphoma” and “hematopoietic stem cell transplantation” or “transplantation” or “therapy” or “treatment.”

### Study Selection

Studies were considered eligible in the analysis if they met the following inclusion criteria: (1) patients were diagnosed with ENKL according to REAL and WHO classifications; (2) patients did not suffer from a second primary malignancy before or together with ENKL; (3) patients were treated with allo-HSCT; (4) studies provided data including overall survival (OS), progression-free survival (PFS), and/or DFS or other markers describing the survival outcome.

Studies that met any of the following criteria were excluded: (1) non-English literature; (2) repeated studies; (3) studies without relevant outcome indicators.

### Data Extraction

Data were extracted by two independent authors and checked by another. Disagreements were resolved in consultation with the third author. For each study, the following information was extracted: (1) basic information such as the author, date, country, patient numbers and characteristics, etc.; (2) specific intervention details; (3) outcome indicators and measures such as OS, PFS, and DFS.

### Data Analysis

The measurement data were expressed in percentages and composition ratios and then the “average age” and “average follow-up time” after allo-HSCT were calculated. The “average age” is only an average value of the “referred ages” of all the references. “Referred age” here means the “mean age” or “median age” in different papers, which are not identical. Theoretically, if all references calculate “mean age” of patients in their case studies, our “average value” of their “referred ages” in this study should equal the mean age of the 136 patients. However, some references calculated “median age” and we had no opportunity to obtain their original data. Thus, we had to take the “median ages” as approximate indicators of the mean ages and finally calculate an average value of those indicators. For instance, if we had collected m references, and if in publication i totally *N_i_*patients were reported, and if the paper said the average age (or mean age, or median age) of the *N_i_* patients was *A_i_*, then the “average age” A of all the patients in the m references selected in this study was calculated as follows:

$A=\frac{\sum_{i=1}^{m}N_{i}A_{i}}{\sum_{i=1}^{m}N_{i}}$

The calculation for “average follow-up time” basically follows the same idea as that for “average age.” In this study, we only calculated these two “average” values. The percentages (for sex, clinical stage, etc.) in Table 1 and Supplementary Table 1 were all calculated using the original counts from the references.

## Results

### Literature Review and Selection

We searched among the articles available in PubMed, Medline, and Embase. Only 17 eligible articles reporting 136 patients were found in this analysis. The male/female ratio of the 136 patients was 1.6:1 and the average age was 40 years (Supplementary Table 1). The detailed clinical features of the 136 patients are listed in Table 2 and Supplementary Table 1.

### Status and Treatment before Allo-HSCT

At initial diagnosis, 38.2% (52) of the 136 patients had early-stage (I/II) disease, 39.7% (54) had advanced (III/IV) disease, and the staging of the other 22.1% (30) was unknown. When receiving allo-HSCT, 46.3% (63) were in CR without disease, 46.3% (63) had residual/refractory/relapsed disease, and the status of the other 7.4% (10) was unknown. Among the 63 patients who were in CR before allo-HSCT, 58.7% (37) were in CR1, 39.7% (25) were in CR2, and the status of the other 1.6% (1) was unknown. Before allo-HSCT, 39.7% (54) of the 136 patients received L-asparaginase-containing chemotherapy and 35.3% (48) received radiotherapy (Table 1).

The 136 patients received a total of 137 successful allo-HSCTs without any graft failure, including one patient who underwent the procedure twice [10]. The patient who underwent treatment twice first received an allogeneic peripheral blood stem cell transplantation (allo-PBSCT) from an HLA-identical donor (younger brother) after myeloablative pretreatment, but 2 months later, he relapsed and had a lymphoma-associated hemophagocytic syndrome. He then received salvage SMILE chemotherapy and high-dose intravenous methylprednisolone; thus, he underwent the second allo-PBSCT from a haplo-identical donor (son) after non-myeloablative (reduced-intensity) pretreatment. Unfortunately, his disease still progressed, and he died of multiple-organ failure [10]. Among the 137 allo-HSCTs, 55.5% (76) of the donors were matched-related, 23.4% (32) were matched-unrelated, 10.2% (14) were haploidentical-related, 4.4% (6) were umbilical cord blood transplantations, and the other 6.6% (9) were unknown.

Among the 136 patients, before allo-HSCT 55.5% (76) received myeloablative conditioning regimens, 42.3% (58) received reduced-intensity conditioning regimens, and the regimens of the other 2.2% (3) were unknown. As for the source of the hematopoietic stem cells, 72.3% (99) were from peripheral blood stem cells, 16.8% (23) were from bone marrow, and 10.2% (14) were from cord blood, while the other 0.7% (1) was unknown.

### GVHD

GVHD prophylaxis for these 136 patients included calcineurin inhibitor (cyclosporine A or tacrolimus), mycophenolate mofetil, short-course methotrexate, and their combinations. At the average follow-up time of 34 months after allo-HSCT, 51 patients (37.5%) had developed acute GVHD and 43 patients (31.6%) had developed chronic GVHD. As the reviewed studies reported, most of these were cases of mild GVHD (grade I or II) (Table 1 and Supplementary Table 1).

### Survival

At the time of the publication of the reviewed studies, 48 patients (35.3%) had relapsed and 70 patients (51.5%) had died. Two of the 48 patients with relapsed ENKL were locally relapsed. One patient received additional irradiation, a course of chemotherapy, and donor lymphocyte infusions [11], while the other one [12] relapsed when her cyclosporine dose was rapidly tapered and discontinued from 80 mg/day, and lesions regressed gradually. These 2 patients achieved and maintained CR again and survived for one additional year. Causes of death included primary disease progression in 30 patients (42.9%), infection in 14 patients (20.0%), GVHD in 8 patients (11.4%), organ failure in 5 patients (7.1%), hemorrhage in 3 patients (4.3%), and other unknown reasons in 10 patients (14.3%) (Table 1).

## Discussion

ENKL is a unique clinical entity characterized by an aggressive clinical course. Both the strategy and the outcome of its treatment depend on disease stage. For patients at lower stages (I/II), combined chemotherapy and radiation therapy is recommended, which achieves a 5-year OS rate ranging from 42% to 83% [13]. In contrast, patients at advanced stages (III/IV) have not been shown to benefit from the addition of radiotherapy and systemic chemotherapy. The overall prognosis of ENKL is poor and its expected 5-year OS is less than 20% [14]. It must also be noted that, although L-asparaginase-based regimens have shown particularly high activity in ENKL patients, a significant number of ENKL patients still relapse after primary therapy, even with the significant toxicity of these regimens. Therefore, people still have substantial interest in alternate treatment strategies [3,15,16,17]. Currently, autologous hematopoietic stem cell transplantation is commonly performed in cases of advanced or relapsed/refractory ENKLs, and allo-HSCT is also reported in some studies.

Allo-HSCT is a potential curative treatment for high-risk lymphoma patients owing to its associated GVL effect. A recent publication by the American Society for Blood and Marrow Transplantation (ASBMT) suggested that allo-HSCT should be limited to advanced-stage and relapsed/refractory ENKL [18]. Recently, several research groups have also investigated the benefit of allo-HSCT in patients with newly diagnosed or relapsed/refractory disease. However, besides case reports, high-quality data on the application of allo-HSCT in ENKL are still limited.

Among the 136 patients reviewed in this study, more patients were middle-aged males with advanced-stage ENKL. Before allo-HSCT, only 39.7% (54) of the 136 patients received L-asparaginase-containing chemotherapy; 35.3% (48) received radiotherapy; 46.3% were in CR (CR1: 27.2% + CR2: 18.4% + unknown: 0.7%) and another 46.3% were in partial remission (PR) or had refractory/relapsed disease. More patients received myeloablative conditioning regimens, and more donors for patients receiving allo-HSCT were matched-related. As for the source of hematopoietic stem cells, more were from peripheral blood stem cells. GVHD is unavoidable in allo-HSCT, but, fortunately, most cases were mild GVHD, such as grade I/II. Finally, the calculated overall mortality for the studies in Table 1 was 51.5%, while in a recent large-scale study of 82 ENKL patients, the mortality rate was as high as 63.4% [19]. The most common reason for death was primary disease progression, still attributed to the highly malignant and aggressive tumor cells.

Clinically, ENKL is characterized by a predominance in young males, is seen in a large proportion as localized stage I and II disease, is refractory to conventional chemotherapy, and is sensitive to radiotherapy [20]. The reported OS varies widely between series. It is known that even limited-stage ENKL has a poor prognosis. The 5-year OS rates with CHOP and involved-field radiotherapy in cases of local nasal ENKL were reported as <50% [21,22]. Some chemotherapy regimens containing L-asparaginase or pegaspargase, such as SMILE, AspaMetDex, and GELOX, have exhibited promising responses. A multicenter analysis from the Asia Lymphoma Study Group [23] reported that patients who had received SMILE chemotherapy before allo-HSCT had significantly better OS and PFS than patients treated with other regimens. However, another study [24] showed that L-asparaginase-containing and L-asparaginase-lacking regimens did not yield a significant difference in 2-year OS and PFS for advanced-stage ENKL, while radiotherapy was associated with significantly prolonged survival in OS and PFS in subgroup analysis. As for the 136 patients reviewed in this study, only 39.7% (54) of them received L-asparaginase-containing chemotherapy, while 35.3% (48) received radiotherapy. Thus, if more patients receive L-asparaginase-containing chemotherapy and radiotherapy before allo-HSCT, it may influence the prognosis, but this needs more verification.

A recent retrospective study from Japan reported the limitations of the new therapeutic strategies and novel regimens, finding almost no improvement in early disease progression and 1-year PFS, while approximately 20% of patients died or experienced disease relapse [25]. Even worse, some studies reported the relapse rate to be as high as nearly 30% in early-stage ENKL [26,27]. Once an aggressive ENKL tumor develops outside the original site, it can grow rapidly and even disseminate with fever, hemophagocytic syndrome, or disseminated intravascular coagulation. Some neoplasms are also multidrug-resistant, leading to relapsed/refractory cases and seriously affecting the prognosis. Therefore, the prognosis of advanced and relapsed/refractory ENKL is poor and the mortality is high. In this study, the overall mortality rate of the 136 patients was 51.5% and the main reason for mortality was primary disease progression. There are two main explanations for this mortality. First of all, although the proportions of advanced-stage and early-stage disease were close (39.7% vs. 38.2%), the other 22.1% of cases (unknown disease stage) may also have included some advanced-stage patients. Second, some patients had refractory/relapsed disease before allo-HSCT with poor prognosis. In such cases, the disease is more likely to progress and even lead to death after allo-HSCT. The second leading cause of death after allo-HSCT was infection, followed by GVHD, organ failure, and hemorrhage. Infection and GVHD are still two major causes of death after allo-HSCT for hematological malignancies. The high treatment-related mortality limits its widespread use.

The prognosis of ENKL is relatively poor. Most diagnosed patients survived <2 years. It was reported that the overall response rate (ORR) after conventional therapy was 36% for newly diagnosed stage IV ENKL and was <10% for relapsed/refractory ENKL [28]. In this study of 136 patients, the ORR was 48.5%, higher than that seen among advanced (or relapsed/refractory) ENKL patients not receiving allo-HSCT. The longest time for maintaining CR after allo-HSCT for these 136 patients was 2617 days. In the follow-up period, only 33.8% (46) of the 136 patients failed to achieve CR after allo-HSCT, but 35.3% (48) relapsed again after allo-HSCT. A multicenter analysis from the Asia Lymphoma Study Group reported that the 5-year OS was 57% and the 5-year PFS was 51% [23]. A large-scale study reported that no transplant recipient had relapsed in 2 years after transplantation, suggesting potent GVL effects [19]. Therefore, allo-HSCT is still a viable therapy option for a subset of ENKL patients. The most important thing is to reduce the high treatment-related mortality.

However, obviously, the toxicity of the pre-allo-HSCT conditioning regimen and possibly the acute post-allo-HSCT GVHD lead to more deaths. A combined analysis [6] of 28 NK-cell neoplasms patients (22 with ENKL) showed that treatment-related mortality was higher in patients receiving conventional myeloablative stem cell transplantation (30% vs. 20%) than that in patients receiving reduced-intensity stem cell transplantation, while the ORRs of the two patient groups had no significant difference (60% vs. 52%). However, non-myeloablative conditioning regimens may lead to high relapse rates, GVHD, and non-engraftment, so the role of non-myeloablative HSCT remains undefined. As the GVL effect has not been definitively established for NK-cell lymphoma, the use of non-myeloablative conditioning should be reserved for patients unsuitable for myeloablative regimens in the setting of clinical trials [29]. In these 136 patients, 72.3% of 137 allo-HSCTs were peripheral HSCTs, perhaps because of collection convenience and the therapy effect being as good as that of bone marrow transplantation. While 10.2% of these 137 allo-HSCTs were cord blood HSCTs, the applicability of umbilical cord blood HSCT needs to be further defined.

The occurrence of GVHD during allo-HSCT cannot be completely avoided. It was reported [23] that the development of acute GVHD had a significant negative impact on OS but not on PFS. Chronic GVHD had an insignificant impact on survival in univariate analysis, while in multivariate analysis, acute GVHD was no longer a significant factor for OS and PFS. In this study, it has been seen that 37.5% (51) of the 136 patients had acute GVHD and 31.6% (43) had chronic GVHD, but the GVHD-induced mortality rate was 11.4%, indicating that the GVHD was generally controllable.

In the authors’ own department, we have treated more than 100 ENKL patients, but only 2 such patients underwent allo-HSCT in the past 5 years. Both of those patients were in an advanced stage (IV) of disease. Of the 2 patients, a female patient had PR after receiving CHOP chemotherapy, then achieved CR after treatment with SMILE regimens. Three months later, she underwent allo-PBSCT from an HLA-related sibling. The IBUCY conditioning regimen was used (idarubicin at 12 mg/m^2^ on days -11 to -9, busulfan at 0.8 mg/kg q6h on days -6 to -4, cyclophosphamide at 50 mg/kg on days -3 and -2). She maintained CR and survived for more than 66 months without any acute or chronic GVHD. The second patient, male, still remained in PR after 4 courses of GLIDE (gemcitabine, ifosfamide, etoposide, dexamethasone, pegaspargase) + methotrexate, and then he received allo-PBSCT from an HLA-related brother, conditioned with FLAG + BUCY (fludarabine at 30 mg/m^2^ on days -10 to -7, cytarabine at 2 g/m^2^ on days -10 to -7, busulfan at 0.8 mg/kg q6h on days -6 to -4, cyclophosphamide at 50 mg/kg on days -3 and -2). He has now been well for 50 months in CR without acute or chronic GVHD.

## Limitations and Conclusion

Due to the rarity of ENKL and the difficulty of conducting randomized controlled trials, the optimal treatment regimen of ENKL has not yet been determined. Consequently, a heavy selection bias might exist. In patients from isolated case reports, a reporting bias toward successful treatment outcomes is apparent. Finally, as none of the studies included in this study were controlled, the outcome of comparable patients treated with conventional chemotherapy and radiotherapy was not available to judge whether allo-HSCT had affected the outcome. Some important information (like remission status, chemosensitivity, overall survival, Kaplan-Meier analysis, etc.) was not elaborate enough in the available references, and this is a shortcoming of the present study. Based on this limited information, we can conclude that allo-HSCT can be considered an option for ENKL, but it should be limited to cases of advanced or relapsed/refractory ENKL. Novel treatments such as anti-CD30 antibody [30] and programmed death protein ligand 1 [31] were reported to be feasible choices for relapsed/refractory ENKL, which have been promising in ongoing trials to confirm their therapy results and help discover combination therapies.

## Figures and Tables

**Table 1:**
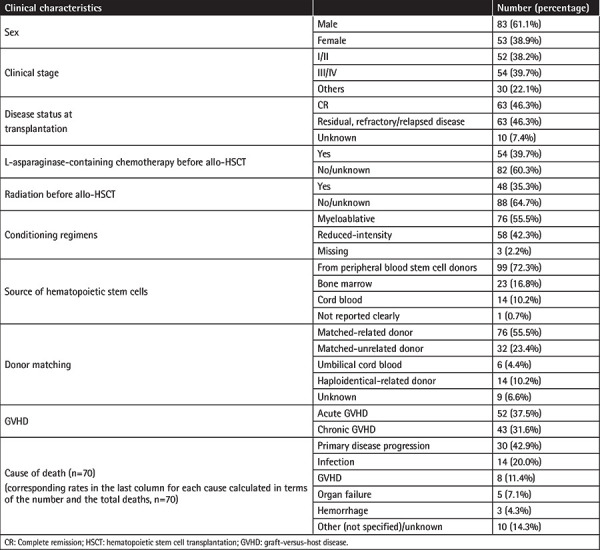
The clinical features of the 136 patients.

**Table 2:**
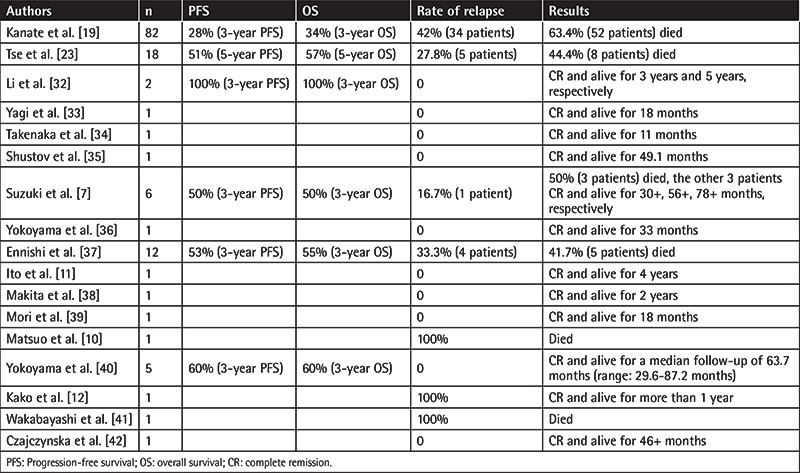
The reviewed studies on 136 ENKL patients undergoing allo-HSCT.
